# Low-Frequency Error Extraction and Compensation for Attitude Measurements from STECE Star Tracker

**DOI:** 10.3390/s16101669

**Published:** 2016-10-12

**Authors:** Yuwang Lai, Defeng Gu, Junhong Liu, Wenping Li, Dongyun Yi

**Affiliations:** 1 Xichang Satellite Launch Center, Xichang 615000, China; laiyuwangchina@163.com; 2 School of Science, National University of Defense Technology, Changsha 410073, China; liujunhongt@163.com (J.L.); dongyunyi@nudt.edu.cn (D.Y.); 3 Beijing Institute of Tracking and Telecommunication Technology, Beijing 100094, China; lwp8422@outlook.com

**Keywords:** star tracker, low frequency error, attitude determination, Vondrak filter, Fourier analysis

## Abstract

The low frequency errors (LFE) of star trackers are the most penalizing errors for high-accuracy satellite attitude determination. Two test star trackers- have been mounted on the Space Technology Experiment and Climate Exploration (STECE) satellite, a small satellite mission developed by China. To extract and compensate the LFE of the attitude measurements for the two test star trackers, a new approach, called Fourier analysis, combined with the Vondrak filter method (FAVF) is proposed in this paper. Firstly, the LFE of the two test star trackers’ attitude measurements are analyzed and extracted by the FAVF method. The remarkable orbital reproducibility features are found in both of the two test star trackers’ attitude measurements. Then, by using the reproducibility feature of the LFE, the two star trackers’ LFE patterns are estimated effectively. Finally, based on the actual LFE pattern results, this paper presents a new LFE compensation strategy. The validity and effectiveness of the proposed LFE compensation algorithm is demonstrated by the significant improvement in the consistency between the two test star trackers. The root mean square (RMS) of the relative Euler angle residuals are reduced from [27.95′′, 25.14′′, 82.43′′], 3σ to [16.12′′, 15.89′′, 53.27′′], 3σ.

## 1. Introduction

High precision attitude determination is very important for many satellite missions [1,2]. The extended Kalman filter (EKF) has been widely used in high precision attitude determination systems consisting of star trackers and rate gyros in the past few decades [3]. Star trackers are optical sensors which measure the angles between stars in order to determine the absolute 3-axes attitude. Star trackers typically yield accuracies an order of magnitude better than other attitude sensors such as an infrared earth sensor, sun sensor and magnetometer, etc. [4,5]. At present, there are mainly two kinds of star trackers, those based on charge coupled device (CCD) detectors, and those based on complementary metal oxide semiconductor (CMOS) active pixel sensor (APS) detectors [6]. To boost the technology development of the star trackers, a new CCD based star tracker (CCD01) and a new CMOS APS based star tracker (APS01) have been loaded on the Space Technology Experiment and Climate Exploration (STECE) satellite as the test payloads. 

STECE, a small satellite mission developed by China, was launched into a dusk–dawn sun-synchronous orbit on 20 November 2011. Its altitude is about 790 km and its orbit inclination is about 98.4°. The predicted life time of STECE is two years (it is still in orbit by now). The STECE satellite uses the earth-oriented 3-axes stabilized attitude determination and control mode, i.e., maintains a fixed-pointing to the earth with its z-axis. Besides the CCD01 and APS01 star trackers, STECE also carries an ASTRO 10 star tracker (CCD based) [7] which is from Jena-Optronik GmbH as the attitude and orbit control system (AOCS) sensor. In addition, STECE carries two fiber optic gyros (FOG) and a dual-frequency GPS receiver. The three star trackers are aligned with their field of view opposite to the direction of the sun. The installation Euler angle of the ASTRO 10 is [13.74° 115.74° 26.41°] in the “3-1-2” sequence (i.e., the yaw–roll–pitch sequence, is used in the whole study), the relative installation Euler angle of CCD01 and APS01 relative to ASTRO 10 is [130.45° −173.81° −48.11°] and [31.29° 81.54° 43.89°], respectively. The boresights of the three star trackers are their own z-axis, respectively. The field of view (FOV) of the CCD01 and APS01 are 20° × 20° and 18° × 18° square, respectively. During the mission, the CCD01 and APS01 provide 10 Hz and 8 Hz quaternion data (represented by [*q*_0_ *q*_1_ *q*_2_ *q*_3_]^T^, a four element vector with *q*_0_ as scalar part and *q*_1_, *q*, *q*_3_ as vector part, which satisfies $q_{0}^{2}+q_{1}^{2}+q_{2}^{2}+q_{3}^{2}=1$).

In the EKF, the star tracker measurement error is generally considered to be Gaussian white noise [8]. However, the measurement of the star tracker contains several error sources which include bias, low frequency errors (LFE) [9,10] and noise. The bias is characterized by a fix offset between the measurement reference frame and the mechanical reference frame. The LFE are errors that vary periodically with the satellite orbit and are of systematic nature. Possible sources of LFE are the thermal effects and changing FOV [11,12]. As the satellite flies along the orbit, the sun irradiation angle varies periodically. Thereby the thermal effect on the satellite platform is not uniform, and this will cause thermal deformation on the optical head and the alignment structure of the star tracker, which yields pointing change of the boresight. In addition, the star tracker’s FOV varies periodically along with the orbit, which causes the number and brightness of the tracked stars to change periodically. Consequently, the star tracker error will fluctuate dynamically. 

Generally, the bias can be eliminated by in-flight calibration algorithms and the noise can be smoothed by filter algorithms [10]. However, the LFE is difficult to eliminate by the in-flight calibration algorithms and also cannot be smoothed by filter algorithms [13,14]. As a consequence, the LFE are one of the most penalizing errors for high accuracy satellite attitude determination. Due to the presence of the LFE, the assumption of Gaussian white noise for star tracker measurement is not appropriate and the performance of the attitude determination EKF is no longer optimal.

Two kinds of approaches have been investigated to mitigate the effect of LFE over the past decade. One approach comes from hardware modifications [10,15], which includes optimizing the thermal–mechanical design, choosing appropriate materials and controlling on-orbital temperature, etc. Another approach comes from error calibration at the measurement level [13,14,16]. In these LFE calibration methods, the LFE are essentially estimated by using the measurement of the gyro. However, the performance of these methods depends on the precision of the gyro closely. The LFE estimation result may be contaminated by the gyro drift and gyro noise. 

In this paper, we analyze the LFE of the CCD01 and APS01 star trackers, and propose a novel approach for star tracker LFE extraction and compensation based solely on their attitude measurements.

## 2. Methodology

The main difficulty in the star tracker LFE extraction and compensation is that we don’t have the true attitude of the satellite. So we need to create a reference attitude quaternion which represents the true attitude as accurately as possible. For this purpose, Schmidt et al. [9] use a fifth degree polynomial to fit the measured quaternions over a time period of 2000 s to acquire the reference quaternions. However, the fit function model of the measured quaternions is unknown, so the selection of the polynomial degree may be subjective and rough. Additionally, according to our experience with real data, the polynomial method is only suitable for fitting the data of a short period. When the attitude data set is long (e.g., several consecutive satellite revolutions), selecting an appropriate polynomial degree is difficult, because the star tracker LFE vary periodically with the satellite orbit (as we demonstrate later in this article), and we need to analyze the attitude data for several consecutive satellite revolutions. Under the condition that the fit function model is unknown and there is a long period of data to analyze, the Vondrak filter may be an appropriate method due to its superior performance [17,18,19]. As for additional benefit, The Vondrak filter relies only on the observed data and the filter values at the two ends of the data series can be calculated. It can expediently and reasonably smooth the data serials by choosing a factor to control its level of smoothing. In this paper, we will use a Fourier analysis combined with a Vondrak filter to fit the star tracker measured quaternion data to obtain the reference quaternion data. For convenience of reference, we will name the Fourier analysis method combined with the Vondrak filter as the FAVF method.

For a series of observational data ($x_{i},y_{i}$), i=1,2,⋅⋅⋅,N, where $x_{i}$ is the measurement epochs and $y_{i}$ is the measurements. The filter values of the Vondrak filter are derived by satisfying the following condition:

(1) $$Q=F+\lambda^{2}S\to \min$$

where F denotes the degree of filtering and S denotes the smoothness of the smoothed curve. The definitions of F and S are as follows:

(2) $$F={\left(N-3\right)}^{-1}\sum_{i=1}^{N}p_{i}{\left(y_{i}-y_{i}^{\prime }\right)}^{2}$$

(3) $$S={\left(s-r\right)}^{-1}\int_{r}^{s}{\left[\varphi^{\prime \prime \prime }(x)\right]}^{2}dx$$

where $y_{i}^{\prime }=\varphi (x_{i})$ is the filter value; $p_{i}$ is the weight of measurements; φ is the smoothed curve expressed in terms of x and $\varphi^{\prime \prime \prime }$ the third numerical derivatives of the smoothed curve which is calculated based on a cubic Lagrange polynomial; $s=x_{N-1}$, $r=x_{2}$. The coefficient $\lambda^{2}$ is a positive coefficient that controls the degree of compromise between the two extreme possibilities: if $\lambda^{2}\to \infty$, S→0 and F→min, the result is a smooth parabola and the operation is called absolute smoothing. If $\lambda^{2}=0$, F→0, then the solution is simply $y_{i}^{\prime }=y_{i}$ and the operation is called absolute fitting. Here $\varepsilon =1/\lambda^{2}$ is called the smoothing factor. 

How to choose the smoothing factor *ε* is the key issue in using the Vondrak filter. A general way is to select the smoothing factor by trial and error. In some cases, the smoothing factor chosen by this way can also meet the needs of the data processing, though it is not the optimal one. A more rigorous way is using the cross-validation method [20,21,22] to find the optimal smoothing factor. The basic concept of the cross-validation method is to divide the observation data into two parts: the filtering series and the validation series. The filtering series is used to generate the filtered results (the smoothed curve) and the validation series is used to cross-validate the filtered results. First, the observation data ($x_{i},y_{i}$) is randomly divided into the filtering part ($x_{1,i}$, $y_{1,i}$), $i=1,2,\cdot \cdot \cdot ,N_{1}$, and the validation ($x_{2,i}$, $y_{2,i}$), $i=1,2,\cdot \cdot \cdot ,N_{2}$ part. Here the filtering sample size $N_{1}$ is much larger than the validation sample size $N_{2}$ in order to maintain the low-frequency signals in the observation data and not to degrade the resolution. Second, for a given smoothing factor $\varepsilon_{l}$, the smoothed curve φ can be obtained based on the filtering part ($x_{1,i}$, $y_{1,i}$). Then, the variance of the validation series relative to the filter values can be calculated by

(4) $$C(\varepsilon_{l},D_{m})=\frac{1}{N_{2}}\sum_{i=1}^{N_{2}}{\left[y_{2,i}-\varphi (x_{2,i})\right]}^{2}$$

where $D_{m}$ denotes the *m*th division of the measurement. For each smoothing factor $\varepsilon_{l}$, suppose that the measurement data is randomly sampled for *M* times, then *M* variances $C(\varepsilon_{l},D_{m})$ can be obtained and the mean of them is acquired by

(5) $$\bar{C}(\varepsilon_{l},D)=\frac{1}{M}\sum_{m=1}^{M}C(\varepsilon_{l},D_{m})$$

Finally, the optimum smoothing factor is the one that makes the $\bar{C}(\varepsilon_{l},D)$ smallest. 

When using the cross-validation method to find the optimum smoothing factor of the Vondrak filter, the *L* different alternative smoothing factors $\varepsilon_{l}=10^{-l}$ (l=1,2,⋅⋅⋅,L) are used to determine the magnitude (supposed as $-\hat{l}$) of the smoothing factor first. Then compare the different smoothing factors $\varepsilon_{k}=k10^{-\hat{l}}$ (usually k=1,2,⋅⋅⋅,9 will be enough) to find the optimum smoothing factor.

Because the STECE satellite maintains an Earth-fixed pointing with its *z*-axis directed towards nadir, as the satellite flies along the orbit, the attitude quaternion measured by the star tracker varies as a sinusoidal wave with a period equal to the orbital period. Therefore, to make the Vondrak filter better fit the quaternion data, we will use the Fourier analysis to extract the large periodic signals of the quaternion data in the FAVF method firstly. Considering that the measured quaternion data are discrete, the periods provided by the Fourier analysis are rough and need to be further adjusted. In this study, the optimal period of the periodic signal with the largest amplitude will be searched around the max period (corresponding to the signal with the largest amplitude) provided by the Fourier analysis. The optimal period will be determined by a performance criterion defined in Equation (7). Then the period signal with the optimal period will be removed from the data. The residuals will be analyzed again by the Fourier analysis to check if there are still periodic signals. The Fourier analysis may be carried out several times until the periodic signal of the residuals becomes negligible.

Let $q_{j,F1}(t_{i},T_{j,1}),q_{j,F2}(t_{i},T_{j,2}),\cdot \cdot \cdot ,q_{j,\mathrm{Fk}}(t_{i},T_{j,k})$ (i=0,1,⋅⋅⋅,N, j=0,1,2,3) be the *k* periodic signals extracted by the Fourier method of the j elements of the measured quaternion $q(t_{i})$ (${\left[q_{0}(t_{i})q_{1}(t_{i})q_{2}(t_{i})q_{3}(t_{i})\right]}^{T}$). Here $T_{j,k}$ is the period of the corresponding periodic signals, N is the size of the data set. Let $\Delta q_{j,Fk}(t_{i},T_{j,k})$ be the corresponding residual, then we have:

(6) $$q_{j}(t_{i})=q_{j,F1}(t_{i},T_{j,1})+q_{j,F2}(t_{i},T_{j,2})+\cdot \cdot \cdot +q_{j,Fk}(t_{i},T_{j,k})+\Delta q_{j,Fk}(t_{i},T_{j,k})$$

The period $T_{j,k}$ is determined by the following performance criterion

(7) $$T_{j,k}=t:\;\underset{t\in [{\tilde{T}}_{j,k}-3,{\tilde{T}}_{j,k}+3]}{\min}J(\Delta q_{j,Fk}(t_{i},t))$$

where ${\tilde{T}}_{j,k}$ is the max period provided by the *k* times Fourier analysis, $J(\Delta q_{j,Fk}(t_{i},t))$ is defined as:

(8) $$\begin{array}{l} J(\Delta q_{j,Fk}(t_{i},t))=\sum_{i=0}^{N}{\left[\Delta q_{j,Fk}(t_{i},t)\right]}^{2}=\sum_{i=0}^{N}{\left[\Delta q_{j,Fk-1}(t_{i},T_{j,k-1})-q_{j,Fk}(t_{i},t)\right]}^{2}\\ =\sum_{i=0}^{N}{\left[q_{j}(t_{i})-q_{j,F1}(t_{i},T_{j,1})-q_{j,F2}(t_{i},T_{j,2})-\cdot \cdot \cdot -q_{j,Fk-1}(t_{i},T_{j,k-1})-q_{j,Fk}(t_{i},t)\right]}^{2} \end{array}$$

When the main periodic signals of the quaternion data have been extracted, the residual $\Delta q_{j,Fk}(t_{i},T_{j,k})$ will be fitted by the Vondrak filter. Let $q_{j,v}(t_{i})$ be the Vondrak filter fit value of $\Delta q_{j,Fk}(t_{i},T_{j,k})$, and $\Delta q_{j,V}(t_{i})$ be the residual error of the Vondrak filter, then we have:

(9) $$\begin{array}{l} \Delta q_{j,Fk}(t_{i},T_{j,n})=q_{j,v}(t_{i})+\Delta q_{j,V}(t_{i})\\ q_{j,v}(t_{i})=\mathrm{Vondrakfit}(\Delta q_{j,Fk}(t_{i},T_{j,k}),\varepsilon ) \end{array}$$

where ε is the smoothing factor of the Vondrak filter.

According to Equations (6) and (9), the final fit value of the j elements is:

(10) $$q_{j,r}(t_{i})=q_{j,F1}(t_{i})+q_{j,F2}(t_{i})+\cdot \cdot \cdot +q_{j,v}(t_{i})$$

Finally, the reference quaternion $q_{r}(t_{i})$ is acquired by

(11) $$q_{r}(t_{i})={\left[q_{0,r}(t_{i})q_{1,r}(t_{i})q_{2,r}(t_{i})q_{3,r}(t_{i})\right]}^{T}/sqrt\left(q_{0,r}{\left(t_{i}\right)}^{2}+q_{1,r}{\left(t_{i}\right)}^{2}+q_{2,r}{\left(t_{i}\right)}^{2}+q_{3,r}{\left(t_{i}\right)}^{2}\right)$$

When the reference quaternion $q_{r}(t_{i})$ is obtained, we can compute the residual quaternions between the reference quaternion $q_{r}(t_{i})$ and the measured quaternion $q(t_{i})$ at the epochs $t_{i}$:

(12) $$\Delta q(t_{i})=q_{r}{\left(t_{i}\right)}^{-1}⊗q(t_{i})$$

where $q_{r}{\left(t_{i}\right)}^{-1}$ is the inverse quaternion of $q_{r}(t_{i})$, defined as $q_{r}{\left(t_{i}\right)}^{-1}={\left[q_{0,r}-q_{1,r}-q_{2,r}-q_{3,r}\right]}^{T}$. The operator ⊗ is quaternion multiplication defined as [23]:

(13) $$q⊗p=\left[\begin{array}{cccc} p_{0} & -p_{1} & -p_{2} & -p_{3}\\ p_{1} & p_{0} & p_{3} & -p_{2}\\ p_{2} & -p_{3} & p_{0} & p_{1}\\ p_{3} & p_{2} & -p_{1} & p_{0} \end{array}\right]\left[\begin{array}{c} q_{0}\\ q_{1}\\ q_{2}\\ q_{3} \end{array}\right]$$

With $q={\left[q_{0}\;q_{1}\;q_{2}\;q_{3}\right]}^{T}$, $p={\left[p_{0}\;p_{1}\;p_{2}\;p_{3}\right]}^{T}$.

The 3-axes attitude measurement residuals are obtained by converting the residual quaternions to the 3-axes delta Euler angles in “3-1-2” rotation sequence:

(14) $$\Delta q(t_{i})={\left[\Delta q_{0}(t_{i})\;\Delta q_{1}(t_{i})\;\Delta q_{2}(t_{i})\;\Delta q_{3}(t_{i})\right]}^{T}\Rightarrow {\left[\Delta \varphi (t_{i})\;\Delta \theta (t_{i})\;\Delta \psi (t_{i})\right]}^{T}$$

where Δφ is the roll angle, Δθ is the pitch angle and Δψ is the yaw angle.

## 3. Results

### 3.1. LFE Extraction Results of the CCD01 and APS 01 Star Trackers

**Figure 1:**
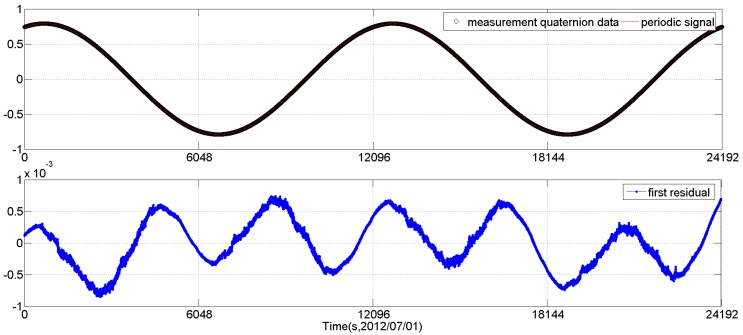
The first pass periodic signal extraction results of *q*_0_ element of CCD01 quaternion data (a sinusoidal signal with a period of 12,096 s is extracted).

**Figure 2:**
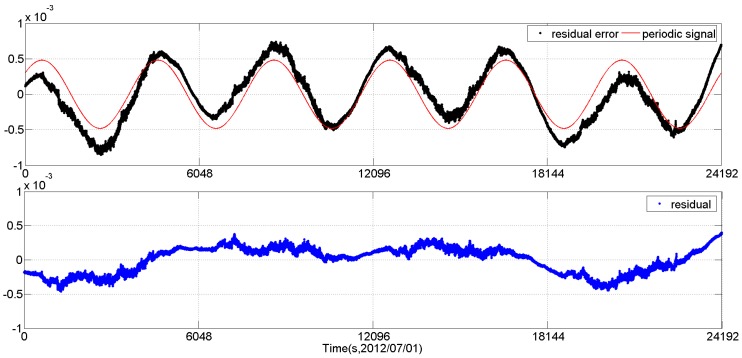
The second pass periodic signal extraction results of *q*_0_ element of CCD01 quaternion data (a sinusoidal signal with a period of 4032 s is extracted).

Figure 1 and Figure 2 show the periodic signal extraction results of the *q*_0_ element of the CCD01 star tracker quaternion data $q(t_{i})$ for four orbits (from 02:13:31 to 08:56:43 of 1 July 2012). In the first pass of the Fourier analysis, a sinusoidal signal with a period of 12,096 s (about two orbits) is extracted. In the second pass, a sinusoidal signal with a period of 4032 s (about 2/3 orbits) is extracted. 

**Figure 3:**
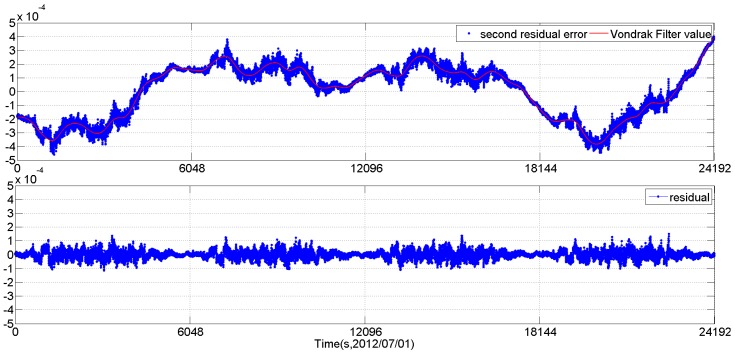
The Vondrak filter results of the *q*_0_ element of CCD01 quaternion data.

We can see that, after the second pass periodic signal extraction, the sinusoidal signal in the residual is not evident any more. At this time, we can use the Vondrak filter to fit the residual. The fitting result is shown in Figure 3. Here, according to the cross-validation method (using 5% of the measurement data series as the validation series and *M* = 50 times divisions), the smoothing factor is selected as 10^−13^.

**Figure 4:**
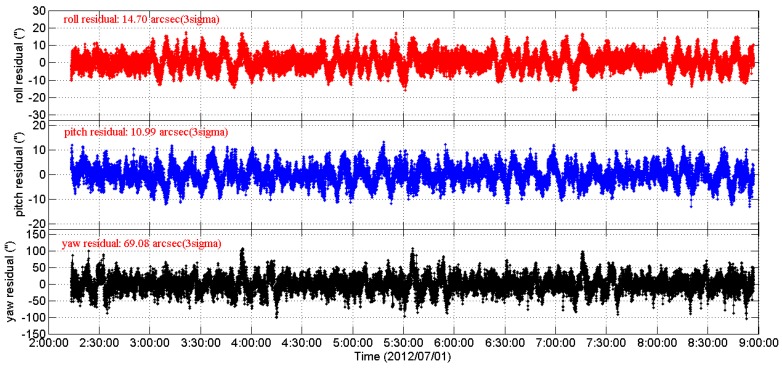
The 3-axes Euler angle residual streams of CCD01 as time series.

**Figure 5:**
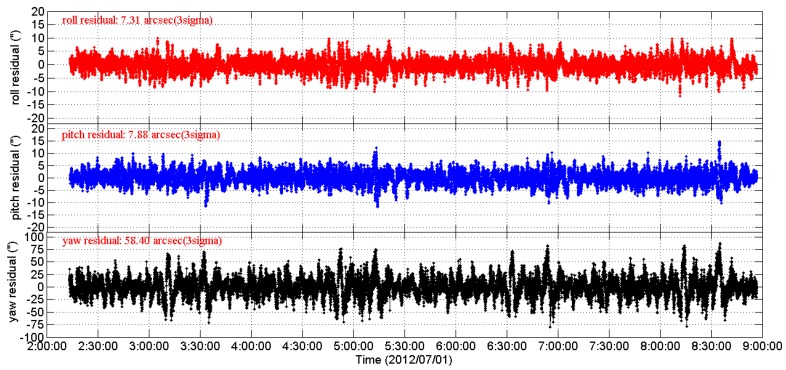
The 3-axes Euler angle residual streams of APS01 as time series.

Figure 4 shows the 3-axes Euler angle residual streams of CCD01 as time series for four consecutive orbits. It can be seen that the 3-axes residual streams contain not only the random noise but also some excursions (drifts and periodic variations which are caused by the LFE). The RMS of the 3-axes residual streams are [14.7′′, 10.99′′, 69.08′′], 3σ. When doing the same data processing of two stage periodic signal extraction plus the Vondrak filter as CCD01, the APS01 star tracker shows similar behavior, as shown in Figure 5.

**Figure 6:**
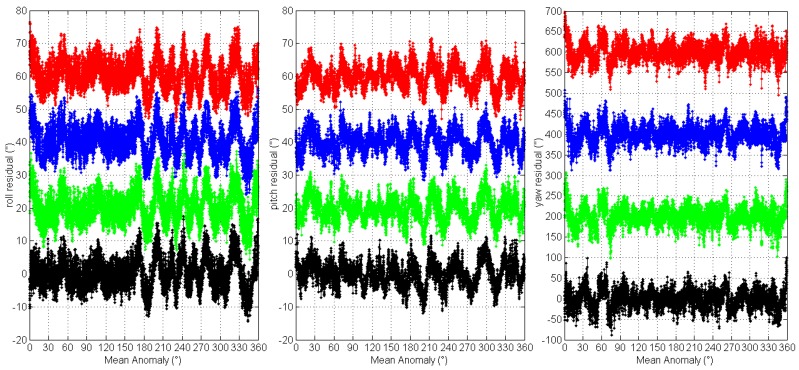
The 3-axes Euler angle residual streams of CCD01 vs. satellite mean anomaly.

**Figure 7:**
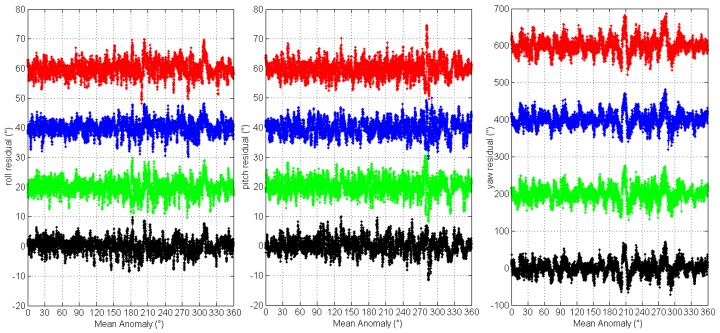
The 3-axes Euler angle residual streams of APS01 vs. satellite mean anomaly.

Further, we compare the four orbits 3-axes Euler angle residual streams of the two star trackers by plotting them versus the satellite mean anomaly (*M*), see Figure 6 and Figure 7. For clarity, the roll, pitch residual streams have been offset by 20′′ and yaw by 200′′ for each orbit. It can be seen that the 3-axes Euler angle residual streams all show the same variations for each star tracker. The excursions repeat from orbit to orbit and features of the residual streams reappear at the same position along the orbit. The orbital reproducibility feature is remarkable. This means that the LFE of the two star trackers are periodic signals with patterns that repeat with each orbital period. 

**Figure 8:**
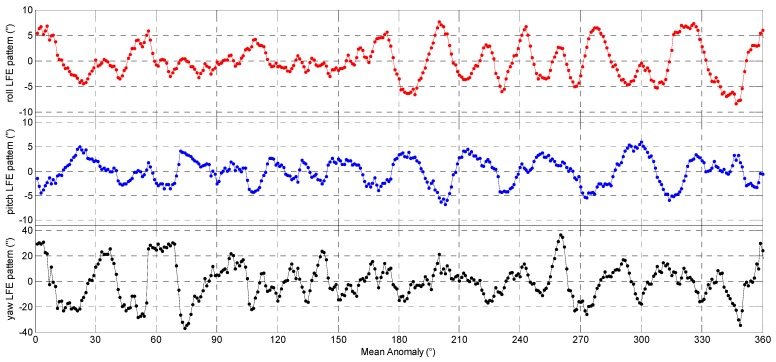
The low frequency errors pattern of the CCD01 star tracker.

**Figure 9:**
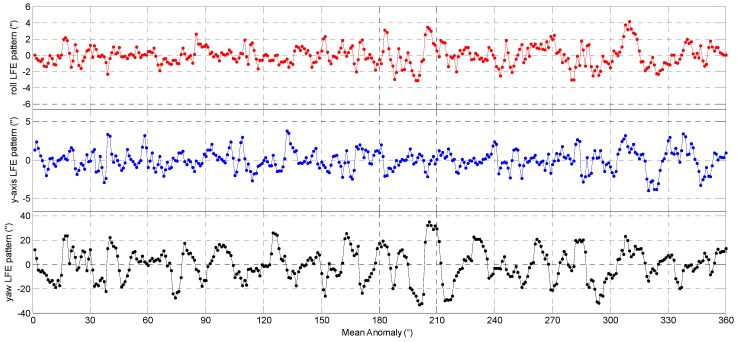
The low frequency errors pattern of the APS01 star tracker.

As the features of the 3-axes delta Euler angles repeat themselves at the same position along the satellite orbit, we can estimate the star tracker LFE pattern as a function of the mean anomaly intervals. In this study, the 3-axes Euler angle error streams are averaged over mean anomaly intervals (with length ΔM). Then, for each interval [(m−1)⋅ΔM,m⋅ΔM) (m=1,2,⋅⋅⋅,360°/ΔM), we will compute the mean value of all the 3-axes Euler angle errors which fall in this range. In this way, the random noise will be smoothed out by multi-orbits statistics and the resulting mean value will be the estimate of the 3-axes LFE. The 3-axes LFE estimation results of the CCD01 and APS01, obtained by using 40 orbit revolutions data from 1 to 3 July 2012, are showed in Figure 8 and Figure 9, respectively. Here the value of Δ*M* is selected as 1°. 

It is important to note that the attitude data used to calculate the LFE pattern cannot be too long because the LFE changes slowly over time. The attitude accuracy of the star trackers depends, among others, on the number and brightness of the stars in the FOV and may be affected by the thermal effects. For a given satellite anomaly, the patterns of stars seen in the FOV are similar for several consecutive satellite revolutions. Furthermore, for the STECE satellite with a dusk-dawn sun-synchronous orbit, the thermal distortion patterns are also similar for several consecutive satellite revolutions. These facts explain the remarkable orbital reproducibility of the LFE. Furthermore, as the earth moves along its orbit around the sun, the stars’ pattern and the thermal distortion pattern (due to different eclipse seasons) will change slowly. As a result, the LFE pattern will change slowly over time too. Similar behavior can also be seen in the star camera systematic errors of the gravity recovery and climate experiment (GRACE) satellite [24,25]. In conclusion, the LFE pattern needs to be calculated again for other periods.

When the 3-axes LFE estimation results (denoted by the 3-axes delta Euler angles $\left[\Delta \hat{\varphi }(m)\;\Delta \hat{\theta }(m)\;\Delta \hat{\psi }(m)\right]$, m=1,2,⋅⋅⋅,360°/ΔM) of the star tracker are obtained, we can remove the LFE from the attitude measurements. Firstly, we convert the LFE estimate delta Euler angles $\left[\Delta \hat{\varphi }(m)\;\Delta \hat{\theta }(m)\;\Delta \hat{\psi }(m)\right]$ to the corresponding delta quaternions (denoted by $\Delta \hat{q}(m)$). Then, according to Equation (12), the LFE of the star tracker attitude measurements are compensated by multiplying the measurement one $q(t_{i})$ with the inverse of the LFE estimate quaternion $\Delta \hat{q}(m)$ if the satellite mean anomaly ($M(t_{i})$) belongs to [(m−1)⋅ΔM,m⋅ΔM):

(15) $$\hat{q}(t_{i})=q(t_{i})⊗\Delta \hat{q}{\left(m\right)}^{-1},\;\mathrm{if}\;M(t_{i})\in [(m-1)\cdot \Delta M,m\cdot \Delta M)$$

### 3.2. LFE Compensation and Validation Results

In the following we are using the relative Euler angle (REA) residual measurements to validate the LFE compensation approach. The REA represents the rotation from one star tracker measurement reference frame to the other star tracker measurement reference frame. The REA residuals are acquired by removing the fixed relative installation Euler angle from the measured REA. Ideally, the REA residuals between two star trackers are constant (ignore the noise) in time. However, the LFE will cause the REA to change with time. Therefore, the LFE compensation approach can be validated by comparing the REA residuals between the CCD01 star tracker and the APS01 star tracker before and after compensating for the LFE. 

**Figure 10:**
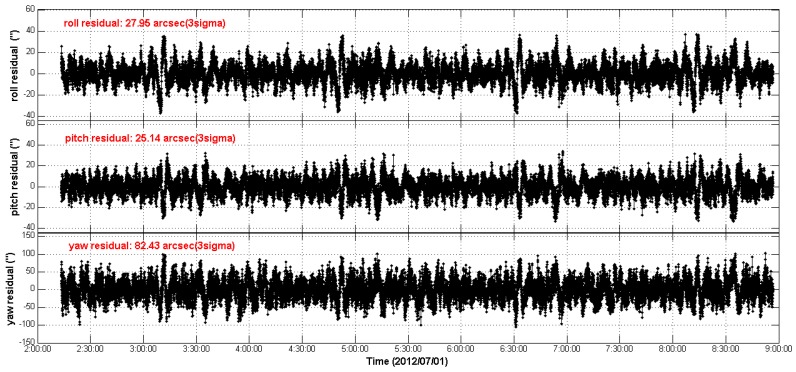
The relative Euler angle residual streams as time series (without LFE compensation).

**Figure 11:**
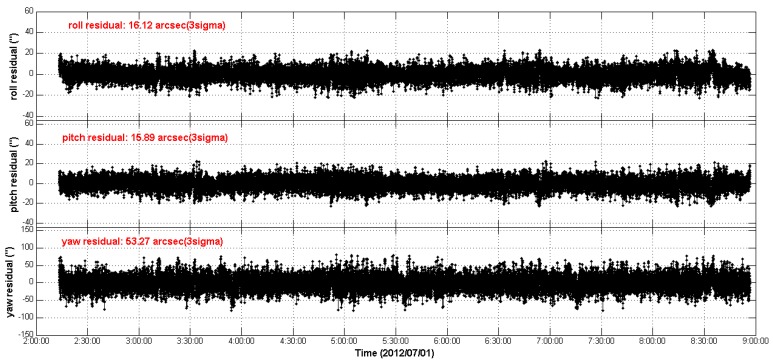
The relative Euler angle residual streams as time series (with LFE compensation).

First, we calculate the REA residuals from CCD01 to APS01 without LFE compensation for the two test star trackers’ quaternion measurements, and the results are shown in Figure 10. It can be seen that, due to the LFE, there are excursions in the REA residual streams and these excursions are repeated from orbit to orbit. The RMS of the 3-axes REA residual streams are [27.95′′, 25.14′′, 82.43′′], 3σ. On the basis of the actual LFE patterns of the two star trackers, we compensate the LFE for the respective quaternion measurements according to Equation (15). The REA residuals with the LFE compensation are shown in Figure 11. As a consequence, the periodic excursions of the REA residual streams in Figure 10 are greatly eliminated and the RMS of the REA residuals are reduced to [16.12′′, 15.89′′, 53.27′′], 3σ. The REA residual streams become more flat as time series. 

**Figure 12:**
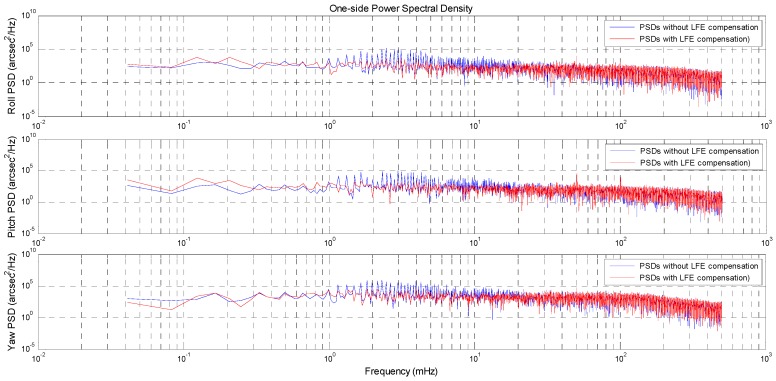
The power spectral densities of the REA residuals (with the x, y-axis in the logarithmic scale).

Figure 12 shows the one-side power spectral densities (PSDs) (with the *x*, *y*-axis in the logarithmic scale) of the 3-axes REA residuals without and with LFE compensation. It can be seen that, before LFE compensation, the PSDs at some low frequencies (in the range of about 1 mHz to 20 mHz) are significantly higher than those at other frequencies. This indicates that the REA residuals have a higher amplitude at these low frequencies. Furthermore, after LFE compensation, the PSDs at these low frequencies have been reduced and the PSDs of the REA residuals become more flat, which indicates that the low frequency components which have the higher amplitude in the REA residuals have been greatly eliminated and the REA residuals are more consistent with white noise. Based on the PSDs of the REA residuals without and with LFE compensation, we can also conclude that the LFE is a strong contributor to the non-white noise in the star tracker data.

In short, both of the REA residual results and their PSDs results demonstrate that the consistency between the two star trackers has improved after the LFE compensation. It is thus clear that the LFE of the two star trackers are compensated effectively and the validity of the proposed attitude LFE compensation approach is demonstrated.

## 4. Summary and Conclusions

This paper presents a new approach to extract and compensate the LFE of the star tracker’s attitude measurement based on the experiment mission of the CCD01 and APS01 star trackers on-board the STECE satellite. Firstly, the LFE of the two test star trackers’ attitude measurements are analyzed and extracted by the proposed approach. By using the Fourier analysis method combined with the Vondrak filter, the remarkable orbital reproducibility feature of the two test star trackers’ attitude LFE is well characterized. Then, the LFE of the test two star trackers are estimated effectively by taking advantage of the LFE orbital reproducibility feature. Finally, based on the actual LFE estimation results, a new LFE compensation strategy is presented.

The significant improvement in the consistency between the two test star trackers’ attitude can be seen by comparison of the REA residual results and their PSDs results before and after LFE compensation. The REA residuals with the LFE compensation become more flat than those without the LFE compensation. The RMS of the REA residual streams are reduced from [27.95′′, 25.14′′, 82.43′′], 3σ to [16.12′′, 15.89′′, 53.27′′], 3σ after LFE compensation.
